# Evaluation of the long-term outcomes of the University of Michigan’s Practice-Oriented Research Training (PORT) program

**DOI:** 10.1017/cts.2023.713

**Published:** 2024-01-18

**Authors:** Phillip A. Ianni, Elias M. Samuels, Brenda L. Eakin, Ellen Champagne, Claire Z. Kalpakjian, Susan L. Murphy

**Affiliations:** 1 Michigan Institute for Clinical and Health Research, University of Michigan, Ann Arbor, MI, USA; 2 Department of Physical Medicine and Rehabilitation, University of Michigan, Ann Arbor, MI, USA

**Keywords:** Continuing education, program evaluation, research design, teaching, healthcare professionals

## Abstract

The University of Michigan created the Practice-Oriented Research Training (PORT) program and implemented it between 2008 and 2018. The PORT program provided research training and funding opportunities for allied healthcare professionals. The program consisted of weekly didactics and group discussion related to topics relevant to developing specific research ideas into projects and funding for a mentored research project for those who submitted a competitive grant application. The goal of this evaluation was to assess the long-term impact of the PORT program on the research careers of the participants. Ninety-two participants (74 staff and 18 faculty) participated in both phases of the program. A mixed-methods approach to evaluation was used; 25 participants who received funding for their research completed surveys, and semi-structured interviews were conducted with eight program participants. In addition, data were collected on participants’ publication history. Fifteen out of the 74 staff participants published 31 first-authored papers after participating in PORT. Twelve out of 15 staff participants who published first-authored papers did so for the first time after participating in the PORT program. Results of quantitative and qualitative analyses suggest that the PORT program had positive impacts on both participants and the research community.

## Introduction

In order to advance translational science, there is a need for efforts on many fronts to identify and break down barriers to the successful conduct of translational research [1]. Healthcare professionals in fields who are involved in implementation but have not been systematically engaged in translational research are an underutilized resource. Healthcare professionals, such as physical and occupational therapy practitioners, typically work as clinicians within academic health centers but usually do not conduct research in that role. Nevertheless, because of their entrenchment in the clinical setting, allied healthcare professionals have the potential to play an innovative role in clinical and translational research [2].

A recent systematic review found that practicing allied healthcare professionals often lack the capabilities, education, and time needed to engage in translational research [3]. For example, although physical therapy practitioners in training in the United States are now required to obtain a Doctor of Physical Therapy degree in order to become a licensed physical therapist [4], and many programs require students to conduct research as part of this degree [5], currently practicing allied healthcare professionals face multiple barriers to conducting research. One important strategy to overcome these barriers identified in the systematic review is to give staff educational opportunities. However, few opportunities are available for currently practicing allied health professionals to receive training that could prepare them to successfully conduct translational research.

Pioneers in filling this gap in training were a group of researchers at the University of Michigan who created the Practice-Oriented Research Training (PORT) program in 2008 to increase the research capacity of allied healthcare professionals [6]. Program participants received first-hand experience in research training by conducting their own research project. In an evaluation of the first year of the PORT program, participants reported significant improvements in their clinical research skills. The present study builds upon this evaluation of short-term outcomes by evaluating the long-term outcomes of the PORT program, which was active between 2008 and 2018.

Previous studies examining the short- and long-term impact of similar programs designed for allied healthcare professionals [7–10] found that participation was associated with several positive long-term outcomes. These included higher research self-confidence, increased enrollment in PhD programs, and submission of publications and conference presentations.

In the present study, we examined several long-term outcomes. First, as evidence that the PORT program improved the research productivity of these trainees, we collected data on the number of manuscripts accepted for publication authored by PORT participants. We also gathered data on participants’ perceptions of their professional development since their participation in the PORT program using self-report surveys and qualitative interviews.

## Methods

### Program design

The PORT program was designed for allied health professionals (including clinical faculty members) who had little or no prior formal training in research. Students participating in graduate programs (i.e., doctoral or master’s level) were ineligible for PORT. Applicants to the PORT program were required to have approval from their supervisor and a departmental agreement to a 50% cost share of up to $5,000 for a research project. Applications were reviewed by PORT program faculty.

Before engaging in research, PORT participants attended a series of didactic seminar sessions, which were intended to help them refine their research ideas and write a study proposal. These sessions addressed topics such as grant writing, protection of human subjects, data collection, research methods, and statistics. Over the 10-year span of the PORT program, several improvements were made. Initially, didactic seminars were presented in two distinct phases, known as Phase 1 and Phase 2 (as described in Murphy et al., 2010) [6]. Phase 1 consisted of three free didactic sessions that were open to all University of Michigan staff and faculty. Interested participants then applied to attend Phase 2, which consisted of nine additional didactic sessions. Over time, the program directors determined that PORT worked best with fewer didactic sessions and benefitted from a flipped classroom approach [11,12]. This required students to prepare more before didactic sessions by reviewing slide presentations; in the didactic session, the presentation was briefly reviewed with a focus on discussion and the topic’s applicability to the projects that were being concurrently developed. In 2013, Phases 1 and 2 were combined and the first three sessions were no longer open to all staff and faculty. Only participants who applied and were admitted to the PORT program could attend the eleven seminar sessions. The final structure of the program in 2018 is described in Table 1.

**Table 1.** PORT timeline (2018)

**January**
PORT application portal is activated
**February**
Applications due
Faculty Directors read and review applications
Faculty Directors provide/finalize admission list and send admission letters
**April–June**
Participants engage in didactic seminars on a weekly basis
Topics covered: Introduction to PORT and Clinical Research, Critical Appraisal of the Literature, Constructing a Literature Review, Specific Aims and Hypotheses, Research Design and Scope, Writing a Research Methods Section, A Gentle Introduction to Power and Statistics, Measurement and Outcomes, Significance and Innovation, Grantsmanship, and Protection of Human Subjects
**October**
Grant applications due
Reviewers recruited and assigned
**November**
Grants reviewed in live study session
Applicants awarded or denied funding
If awarded, applicants address application concerns and re-submit
**December**
Re-submissions due back to PORT faculty and program office
Award letters sent to successful applicants
**January**
Research project phase begins

After completing the 11 didactic sessions of the program, participants were required to submit a grant application to the PORT review committee for a proposed project. Participants began working on their grant application during the didactic phase. Between sessions, participants engaged in homework assignments that consisted of writing different sections of the grant application, which was based on elements of a National Institutes of Health (NIH) R grant application. Applications could be submitted either by individual participants or by teams of participants. A team leader was designated as principal investigator (PI), and other team members were designated as co-investigators (co-I).

The grant application included a project summary, budget, investigator biosketches, mentor biosketch, proposal narrative, and letters of support. The grant applications were reviewed by a team of PORT program faculty and postdoctoral fellows and evaluated on four criteria: research problem formulation, research methodology, scientific writing and grantsmanship, and human subjects protection. Each application was assigned a score and either awarded or denied based on that score. If awarded, participants and teams advanced to the research project stage.

In the research project stage, participants and teams worked with program mentors to carry out their research projects. Each participant received $5,000 to do this, which was typically matched by their home department. Participants were allowed a maximum of two one-hour consultations with the Michigan Institute for Clinical and Health Research (MICHR) Biostatistics unit to obtain statistical support for their projects. Most participants in the research project phase needed two years to complete the program; however, some received no-cost extensions beyond these two years to complete their projects. In order to receive the PORT program’s certificate of completion, participants were required to submit a poster abstract to an annual research conference held at MICHR or any conference of their choosing.

### Termination of the PORT program

The PORT program admitted its final cohort of participants in 2018, and active research teams continued to work on their projects until their funding concluded at the end of 2020. We decided to stop offering the program for two primary reasons. First, there was a university and health system restructuring in some healthcare units, ending their ability to offer protected time for staff to conduct research. Second, after 10 years of evolution of the program to optimally support allied healthcare professionals, we recognized that we had reached a saturation point for our ability to continue to innovate learning in this area. Therefore, we focused our efforts on disseminating the program by developing robust training materials and slides for others to use (as of this writing, these materials are freely available from https://www.diamondportal.org/).

### Evaluation

The present study builds upon the previous short-term evaluation of PORT [6] by examining long-term publication, survey, and interview data. We used mixed methods to evaluate the impact of the PORT program on the ongoing engagement of clinician-trainees in research activities. We used surveys to collect data on participants’ perceptions of the program. Later, we conducted one-on-one interviews to develop a deeper understanding of the survey data. To assess long-term outcomes of the program, we collected data on PI’s and co-I’s subsequent publications from PubMed.

In April 2022 (2–12 years after participants completed their participation in the PORT program), participants who received funding for their research were surveyed to collect data on the long-term impact of the program. The survey was sent to the participant’s email address on file and was administered using the Qualtrics platform [13]. Participants were sent up to three reminders if they did not respond to the initial survey distribution.

The survey, which consisted of a mix of closed yes/no items and open-ended items, asked participants about how the PORT program impacted their career. We decided to use a yes/no response format to lower response burden to participants, given the large number of survey items. These questions were arranged into six components: knowledge and skills, grant awards and patents, publications and presentations, collaborations and networks, professional advancement, and institutional culture and practice. These components were selected based on themes identified by previous research [7–9]. They were also asked whether they were currently involved in health research activities, their satisfaction with their professional career, and how their career has been different because of the PORT program.

Survey participants who indicated that they were interested in talking more about how their participation in the PORT program contributed to their careers were contacted about participating in a 30-minute interview. Interviews were conducted in November 2022 with eight program participants. Interviews were semi-structured and tailored to the specific participant being interviewed based on their survey responses, with the goal being to fill in gaps in the information and to get a deeper understanding of the participants’ responses to the survey. Participants were asked several questions, including why they got involved with the program, what institutional supports/services enabled them to participate in PORT, what was the most impactful aspect of PORT, how participating in PORT contributed to their career, what short-term and long-term impacts participating in PORT had on their professional work, why they would recommend participating in PORT to other researchers, the most significant barriers that other researchers would face in trying to participate in the program, and any potential improvements to PORT.

## Results

### Program participants

Between 2008 and 2018, 64 team applications were submitted for review. Forty-eight of these applications were funded (two declined); 16 were denied funding. Seventy-four allied healthcare professionals and eighteen faculty participated in the research phase of the PORT program, either as a PI or Co-I on a proposal (one staff member participant later became a faculty member after participating in PORT; they are counted as staff in this paper). The number of teams and individuals participating in each cohort year is shown in Table 2. Team sizes averaged two people, with a range of 1–4.

**Table 2.** Number of team applications and funded teams by cohort year

Cohort	Team applications	Denied applications	Declined	Funded teams	Total individuals on funded teams (n)	Allied health professionals
2008–10	4	0	0	4	9	9
2009–11	8	1	0	7	19	11
2010–12	12	5	0	7	13	10
2011–13	5	2	0	3	9	9
2012–14	3	0	0	3	4	3
2013–15	6	0	0	6	8	5
2014–16	4	0	0	4	6	6
2015–17	6	1	0	5	7	4
2016–18	8	5	1	2	5	5
2017–19	6	2	0	4	9	9
2018–20	2	0	1	1	3	3
Total	64	16	2	46	92	74

The composition of program participants changed over time. The program was initially designed for physical therapy and occupational therapy practitioners in the University of Michigan Department of Physical Medicine and Rehabilitation [6]. Over time, enrollment expanded to include other allied healthcare professionals (e.g., audiologists, orthotists, nurse practitioners, clinical social workers) and clinical faculty interested in the program. Clinical faculty tended to serve on teams with other clinical faculty members, rather than leading teams of staff. For this evaluation, we excluded outcomes data on clinical faculty members who participated in PORT. We chose to exclude faculty because they typically had a research career before their participation in PORT.

Participants had a variety of professional roles. The most common were physical therapist (*n* = 22), followed by occupational therapist (*n* = 15), clinical social worker (*n* = 12), clinical assistant professor (*n* = 7), clinical instructor (*n* = 6), and nurse practitioner (*n* = 3). The remaining twenty-seven participants fell into a job category with two or fewer participants.

### Publications

For this analysis, we included only articles that were: 1) published after the participant started the research phase of the PORT program, 2) published by University of Michigan staff members rather than faculty, and 3) those for which the PORT participant was first author. We focused on publications where the participant was first author because it is likely that the PORT participant led the project. There were 154 papers on which staff participants were credited as a coauthor or first author. Fifteen out of 74 staff published one or more first-authored papers after participating in the PORT program, totaling 31 papers. Of these 15, eight were PI’s and seven were co-I’s. Of the 31 first-authored publications, nine (29%) had at least one additional PORT participant serving as a coauthor. Only three of these 15 participants had ever published a paper as a first author before they participated in the program, and twelve of these 15 participants published as a first author for the first time after their participation in PORT.

Publications were analyzed for impact using iCite Bibliometrics [14] and Altmetric data [15]. iCite Bibliometrics were available for all 31 post-PORT first-authored publications. These papers had a total of 263 citations, with a median of 7.0 and a maximum of 44. The average number of citations per year was 1.4 (max = 6.3, median = 1.0). The average Relative Citation Ratio was 0.76 (max = 3.0, median = 0.69). Altmetric data were available for all but one of the staff participants’ post-PORT publications. There were 22 publications with “mentions” indicating attention, with a cumulative total of 152 “mentions.” There were 148 social media mentions, two news/blog mentions, one policy document mention, and one Wikipedia mention. For the 123 publications coauthored by at least one PORT staff participant, the total number of citations was 2,440, with a median of 5.0 citations per publication.

### Survey results

In 2022, the 92 PORT participants who participated in the research phase of the program were asked to complete a survey about their current engagement in health research activities, and 25 responded (Table 3).

**Table 3.** Percentage of participants (*N* = 25) who reported gains as a result of participating in the PORT program

	As a result of participating in the PORT program…	% Yes Responses
**Knowledge and Skills** (average 98%)	… I gained research skills that I have applied to my work.	100%
	… I gained skills relevant to clinical and health research.	100%
	… I built on the skills I gained through PORT with subsequent professional development.	95%
**Institutional Culture and Practice** (average 85%)	… I gained a better understanding of research resources available to me at U-M.	96%
	… I am better able to contribute to a culture of evidence-based practice in healthcare.	92%
	… I am better able to understand how I can contribute to clinical/translational research studies.	92%
	… I gained a better understanding of the research enterprise at U-M.	84%
	… I am better able to integrate appropriate and relevant research findings into my practice.	83%
	… I am better able to problem solve during common clinical or health research experiences.	83%
	… I am better able to keep up with advancements in research.	65%
**Collaborations and Networks** (average 69%)	… I am better able to collaborate or communicate with health research teams.	92%
	… I am better able to collaborate or communicate with healthcare teams.	87%
	… I met new collaborators with whom I have conducted research.	68%
	… I gained access to new professional networks within or outside U-M.	64%
	… I met new collaborators with whom I have worked in a professional capacity other than research.	56%
	… I met new colleagues with whom I have maintained contact.	50%
**Professional Advancement** (average 66%)	… I received any kind of recognition for my professional development.	87%
	… I gained a better understanding of the kind of work that gives me better job satisfaction.	79%
	… I gained a better understanding of my professional goals.	75%
	… I was better able to advance my career goals.	74%
	… I added a reference to my PORT participation on my resume or CV.	68%
	… I was better able to access new opportunities for professional advancement.	54%
	… I received a promotion at work.	22%
**Publications and Presentations** (average 48%)	… I presented clinical or health research at an academic conference.	73%
	… I published clinical or health research in a peer-reviewed journal.	54%
	… I disseminated the results of clinical or health research through other means (please specify).	44%
	… I presented clinical or health research at other public events.	41%
	… I published clinical or health research in non-peer-reviewed journals or as white papers.	29%
**Grant Awards and Patents** (average 45%)	… I received funding for a clinical or health research study.	60%
	… I submitted a proposal to fund a clinical or health research study.	60%
	… I received a patent on a device, technique or idea related to my work in the PORT program.	14%

In the same survey, participants were also asked about their current engagement in health research activities, and 24 responded. Seventeen (81%) indicated that they were currently involved in clinical and translational research, including active membership in a multidisciplinary health research team, 11 (52%) were currently involved in supporting funded clinical and translational research, 12 (57%) were currently involved in project writing/re-submitting/reviewing clinical and translational research, six (29%) were currently involved in directing clinical and translational research projects, and five (24%) were currently involved in funding, conducting, or managing clinical and translational research.

### Qualitative data

To better understand the meaning of our quantitative findings, we conducted interviews with several PORT participants who have successfully published as a first author. Interview transcripts from eight participants were coded by three coauthors (ES, EC, and PI) who have expertise in qualitative analysis. Using the RADaR technique [16], the coders independently identified relevant passages of text that exemplified the six themes identified from the quantitative results. The coders then met to reconcile the assignment of quotes to themes. The six themes and representative quotes are shown in Table 4.

**Table 4.** Themes and sample quotes from interview participants

Theme	Quote
Knowledge and skills	“I kind of think of PORT as almost like a graduate degree, even though I know it wasn’t. But it taught me to think differently, and it taught me how to use the research process, even in sort of thinking about quality improvement things.” “The nice thing about PORT is that it was very targeted… I mean it was down and dirty. It was like: This is the research process. This is the IRB process…if really what you want is skills that you need to start doing research, you know this would be an alternative that is going to be less time, and you don’t have to do a lot of the fluff stuff a lot of my doctoral students complain about.”
Institutional culture and practice	“I think I actually would recommend it to clinicians who are interested in helping more than one person at a time but don’t have a way to address it and research gives you that way to address it.” “The research journey begun with PORT has led to expansion of other projects and work to improve patient-family and staff experience at University of Michigan Health.”
Collaborations and networks	“The mentor that I chose in the department of pediatric neurology, and I became very close and I really enjoyed working with her and getting to know her.” “PORT has given me a solid foundation to be a collaborator and to develop a network.”
Professional advancement	“I don’t think I would have been given those opportunities without having a publication like I did and I wouldn’t have gotten the publication done without PORT.” I never thought that I would leave clinical work to do research, but because of PORT… I could actually look for work in a different area. And I applied and actually got the first job I applied for because of my research background.”
Publications and presentations	“In the project that we did for PORT, we did submit it for publication. It did not get published, and that was actually a good learning experience, because the way that we approached that project taught me a lot about how to approach things differently.” “The biggest [impact of PORT] is that I’ve done 2 poster presentations at national conferences, a presentation at a national conference, and I have 2 publications that I published in national journals.”
Grants and awards	“I then got a 2-year grant through the state…to do additional research, and I became the research lead for the Department for about 5 years.”

These sample participant quotes demonstrate that the program had multiple positive impacts on participants. Participants reported that the PORT program helped them gain knowledge and develop skills quickly and efficiently. By enabling participants to engage in research, the PORT program changed participants’ views of institutional culture, enhanced their work with patients through evidence-based practice, and supported quality improvement in the clinical area. One participant stated that their status as a primary investigator was used as part of a departmental application to obtain Magnet recognition for their department. PORT helped participants establish relationships with mentors and develop a professional network. Participants said that PORT gave them the research background required to get jobs that they would not have qualified for had they not taken the program. Lastly, participants reported that PORT gave them the opportunity to submit manuscripts for publication, present their findings at scientific conferences, and help participants obtain grants to conduct research.

## Discussion

The results of this study demonstrate that the PORT program had a considerable impact on participants. Publications data showed that participants produced more than five times as many manuscripts after participating in PORT than before they entered the program. Survey data and interviews showed that participants believed the PORT program benefitted them greatly, especially on their self-assessed knowledge and skills. Taken together, we interpret this as evidence that our program was successful in giving healthcare professionals much-needed knowledge and skills to lead their own research projects. These findings are consistent with previous studies of similar programs [7–9].

PORT participants were typically not expected to disseminate research as part of their clinical role; however, the PORT program encouraged dissemination of findings and offered mentorship in manuscript writing as needed. This support may have helped bolster the number of publications produced by participants after completing the program. In fact, 12 out of the 15 participants who published manuscripts as first author after participating in PORT had no prior experience writing for publication. The PORT program appeared to have the right support for enabling participants to conduct and lead their own research projects, as well as disseminate their findings. In addition, several participants published with their fellow PORT team members after their participation in the program ended. Nine of the 31 first-authored publications had at least one additional PORT participant serving as a coauthor, demonstrating that this training was successful in catalyzing team science.

### Limitations

While the results of the program appear impactful to this group of healthcare professionals, they must be interpreted with caution. The lack of a comparison group prevents us from being able to directly attribute every first-authored publication to the PORT program, especially those articles written well after participation in the program. Nevertheless, we inferred that all first-authored publications produced after participating in PORT could be attributed at least in part to participants’ involvement in the PORT program as they acquired relevant research skills.

The low response rate to the survey represents another limitation. As only about 30% of PORT participants responded to the survey, they may not have been a representative sample. We believe the response rate was low because we surveyed participants who may have completed their participation in PORT up to 12 years prior to the survey date. During that time, PORT participants may have retired from the university or changed jobs and left the university. While we sent the survey to the participants’ email addresses on file, not all emails were current and some bounced. In addition, those who had published as a first author were more likely to respond to the survey. Therefore, their responses may not represent the sentiment of all participants. However, while our results are highlight experiences from a subset of the entire sample, at least this subset had a positive and impactful experience in PORT that supported their professional careers.

## Conclusion

These data address an important need in clinical and translational research by expanding the research workforce. We recommend academic medical centers use our PORT program materials to create similar programs to support members of their clinical workforce who are integrating research and practice into their careers. We believe that the PORT program, and programs like it, maybe a feasible alternative to graduate school for currently practicing healthcare professionals. Future research is needed to address whether this program could also be successfully exported to other populations in the medical realm, like physicians. Our results suggest that programs like PORT could give healthcare professionals the skills they need to test hypotheses informed by their real-world experience.

## References

[1] Austin CP. Opportunities and challenges in translational science. Clin Transl Sci. 2021;14(5):1629–1647. doi: 10.1111/cts.13055. 33982407 PMC8504824

[2] Arena RA , Goldberg LR , Ingersoll CD , Larsen DS , Shelledy D. Research in the allied health professions: why fund it? A report of the ASAHP Research Committee. J Allied Health. 2011;40(3):161–166. 21927783

[3] Smith S , Johnson G. A systematic review of the barriers, enablers and strategies to embedding translational research within the public hospital system focusing on nursing and allied health professions. PLoS One. 2023;18(2):e0281819. doi: 10.1371/journal.pone.0281819. 36795679 PMC9934318

[4] Commission on Accreditation in Physical Therapy Education. Standards and required elements for accreditation of physical therapist education programs. https://www.capteonline.org/globalassets/capte-docs/capte-pt-standards-required-elements.pdf. Accessed July 27, 2023.

[5] American Council of Academic Physical Therapy. PhD Program Directory Related to Physical Therapy (PT PhD’s). https://acapt.org/about/consortium/research-intensive-programs-physical-therapy/rippt-programs/phd-program-listings. Accessed October 5, 2023.

[6] Murphy SL , Kalpakjian CZ , Mullan PB , Clauw DJ. Development and evaluation of the University of Michigan’s Practice-Oriented Research Training (PORT) Program. Am J Occup Ther. 2010;64(5):796–803. doi: 10.5014/ajot.2010.08161. 21073110

[7] Harding KE , Stephens D , Taylor NF , Chu E , Wilby A. Development and evaluation of an allied health research training scheme. J Allied Health. 2010;39(4):e143–e148. 21184016

[8] Harding KE , Shields N , Whiteside M , Taylor NF. “A great first step into research”: stepping into research is an effective and sustainable model for research training in clinical settings: a report of 6-year outcomes. J Allied Health. 2016;45(3):176–182. 27585613

[9] Harvey D , Barker R , Tynan E. Writing a manuscript for publication: an action research study with allied health practitioners. Focus Health Prof Educ Multi-Discipl J. 2020;21(2):1–16.

[10] Janssen J , Hale L , Mirfin-Veitch B , Harland T. Building the research capacity of clinical physical therapists using a participatory action research approach. Phys Ther. 2013;93(7):923–934. doi: 10.2522/ptj.20120030. 23559527

[11] Hurtubise L , Hall E , Sheridan L , Han H. The flipped classroom in medical education: engaging students to build competency. J Med Educ Curric Dev. 2015;2:JMECD.S23895. doi: 10.4137/JMECD.S23895. 35187252 PMC8855432

[12] Phillips J , Wiesbauer F. The flipped classroom in medical education: a new standard in teaching. Trends Anaesth Crit Care. 2022;42:4–8. 10.1016/j.tacc.2022.01.001 PMC9764229 38620968

[13] Qualtrics. Provo, Utah. https://www.qualtrics.com.

[14] NIH iCite. https://icite.od.nih.gov/. Accessed October 5, 2023.

[15] Altmetric. https://www.altmetric.com/#. Accessed October 5, 2023.

[16] Watkins DC. Rapid and rigorous qualitative data analysis: the “RADaR” technique for applied research. Int J Qual Methods. 2017;16(1):1–9.

